# Measuring Clinical Benefit of Treatments for Hematologic Malignancies: Critical First Steps Accomplished—What is Next?

**DOI:** 10.1097/HS9.0000000000000354

**Published:** 2020-04-01

**Authors:** John G. Gribben, Elisabeth de Vries, Nathan I. Cherny, Pieter Sonneveld

**Affiliations:** 1 Barts Cancer Institute, Queen Mary University of London, London, United Kingdom; 2 European Society for Medical Oncology, Department of Medical Oncology, University Medical Center Groningen, University of Groningen, Groningen, The Netherlands; 3 European Society for Medical Oncology, Department of Medical Oncology and Palliative Medicine, Shaare Zedek Medical Center, Jerusalem, Israel; 4 European Hematology Association, Department of Hematology, and Department of Radiation Oncology, Erasmus MC Cancer Institute, Rotterdam, The Netherlands

Instruments that measure clinical benefit are useful to support policy decision making. They can help to assess the value of new treatments and may provide insights to inform the design of future clinical trials.^1–3^ The European Society for Medical Oncology (ESMO) developed a valuable tool to assess the magnitude of clinical benefit from new cancer therapies – the ESMO-Magnitude of Clinical Benefit Scale (ESMO-MCBS) – that has been validated and proven reproducible in solid tumor oncology.^4,5^ This value framework is currently incorporated into ESMO's clinical practice guidelines and is being used to inform health technology assessment processes.^1,2^

The European Hematology Association (EHA) has observed the development of the ESMO-MCBS with interest, since no oncology frameworks have yet been validated for assessing treatments for hematologic malignancies. Given the increasing number of treatments available for these types of malignancy, that often target small, well-defined patient populations, availability of an appropriate validated assessment for hematologic malignancies is of pressing importance. Consequently, experts from EHA and ESMO tested the latest version of ESMO-MCBS (version 1.1) in several hematologic malignancies, and their findings have recently been published.^6^

Experts from relevant EHA Scientific Working Groups identified and graded 80 studies of recently approved treatments for hematologic malignancies using the ESMO-MCBS version 1.1 forms.^5,6^ Their gradings were then reviewed by the ESMO-MCBS Working Group to ensure applicability and correctness. The hematologic malignancies considered included acute lymphoblastic leukemia (ALL), acute myeloid leukemia (AML), chronic lymphocytic leukemia (CLL), chronic myeloid leukemia (CML), Hodgkin and non-Hodgkin lymphomas, multiple myeloma (MM) and myelodysplastic syndromes (MDS). For each of these diseases, the scoreability of the studies and the reasonableness of the derived scores were evaluated so that any shortcomings in the current version of ESMO-MCBS, when applied to each hematological malignancy, could be identified. Clinical benefit was graded for treatments with curative intent and for treatments with non-curative intent.

The ESMO-MCBS version 1.1 was widely applicable to the overwhelming majority of studies analyzed by EHA experts. Of the 80 studies analyzed, 72 (90%) could be scored, and the scores generated were generally considered to correspond with expert assessment of the magnitude of clinical benefit obtained for the treatments under evaluation. This is encouraging but suggests that more work is needed to increase this further.

A key aim of this project was to probe the ESMO-MCBS more deeply, to identify key differences between sold tumors and hematological malignancies in order to isolate the most important shortcomings that would need to be addressed and amended in order to develop a version that is applicable more robustly to the full spectrum of hematologic malignancies. Due to the high degree of cooperation that existed between the scientific leadership of the ESMO-Magnitude of Clinical Benefit Working Group and the European Hematology Association over the past 2 years, this project has resulted in the completion of this work, which has recently been published in ESMO Open.^6^

Some shortcomings in the capacity of ESMO-MCBS to grade the magnitude of clinical benefit appropriately were identified for almost all types of hematological malignancies (with the exception of AML); these shortcomings are summarized in Table 1.

**Table 1:**
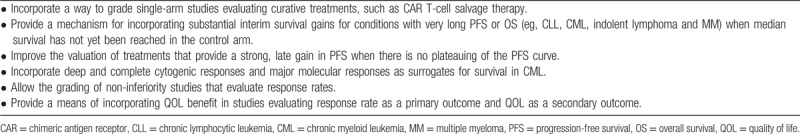
Amendments Identified for ESMO-MCBS Version 1.1 to Incorporate Hematologic Malignancies.

This critical accomplishment is now facilitating the next phase of this ongoing collaboration between our organizations, the development of a robustly validated version of the ESMO-MCBS for hematological malignancies: ESMO-MCBS:H. This process is underway: a draft version of ESMO-MCBS:H has been prepared which will undergo scientific review, statistical validation, extensive field testing and a process of expert peer review for reasonableness. These methodological stringencies are essential to meet the standards of “accountability for reasonableness”^7^ that underlie the integrity of this endeavor which is critical to both EHA and ESMO and to the use of the tool in public health policy.

We look forward to this important challenge with the secure knowledge that we now have an established track record of tremendous cooperation. We believe that the ESMO-MCBS:H will make a major contribution to the word of malignant hematology.

## Conclusions

The ESMO-MCBS version 1.1 is widely applicable to studies of recently approved treatments for hematological malignancies analyzed by EHA experts. However, a number of modifications are necessary to enable hematological malignancies to be validated within the ESMO-MCBS framework. Based on the findings of this EHA analysis, EHA and ESMO are committed to develop a version of the scale that is robustly validated to grade studies of new treatments for hematological malignancies. Collaboration between these organizations is ongoing in order to achieve this in a timely manner.
